# Functional diversity and team innovation: A study on the mediating role of social cohesion in primary care teams

**DOI:** 10.1097/HMR.0000000000000369

**Published:** 2023-03-27

**Authors:** Alissa Lysanne van Zijl, Brenda Vermeeren, Ferry Koster, Bram Steijn

**Affiliations:** **Alissa Lysanne van Zijl, PhD,** is Assistant Professor, Erasmus University Rotterdam, the Netherlands. E-mail: vanzijl@essb.eur.nl.; Brenda Vermeeren, PhD, is Associate Professor, Erasmus University Rotterdam, the Netherlands.; Ferry Koster, PhD, is Professor, Erasmus University Rotterdam, the Netherlands.; Bram Steijn, PhD, is Professor, Erasmus University Rotterdam, the Netherlands.

**Keywords:** Functional diversity, interprofessional teams, primary care, social cohesion, team innovation

## Abstract

Bringing together professionals with different knowledge and skills comes with the opportunity to spur the innovativeness of primary care teams. Nevertheless, empirical evidence shows that it is not self-evident that these innovations are also realized. The social categorization theory suggests that a better understanding of whether these potential team innovations are realized can be obtained by looking at the social cohesion of such teams.

**Purpose:**

The aim of this study was to study the relationship between functional diversity and team innovation in primary care teams by examining the mediating role of social cohesion.

**Methodology:**

Survey responses and administrative data of 887 primary care professionals and 75 supervisors in 100 primary care teams were analyzed. Structural equation modeling was used to examine a curvilinear mediated relationship among functional diversity and team innovation through social cohesion.

**Results:**

The findings show a positive relationship between social cohesion and team innovation as expected. Contrary to the expectations, the relationship between functional diversity and social cohesion is insignificant, and the results show an inverted U-shaped relationship between functional diversity and team innovation instead.

**Conclusions:**

This study reveals an unexpected inverted U-shaped relationship between functional diversity and team innovation. This relationship is not mediated by social cohesion; however, social cohesion is still a significant predictor of team innovation.

**Practice Implications:**

Policymakers should be aware of the relevance as well as the complexity of creating social cohesion in functionally diverse primary care teams. As long as it remains unknown how social cohesion is stimulated in functionally diverse teams, it seems best for the team innovation to prevent bringing together too many, but also too few, different functions.

Functionally diverse teams, also known as interprofessional teams, come with great potential to stimulate innovation by their access to a wide range of knowledge (Mitchell & Boyle, 2021). The use of functionally diverse teams, in which different professional roles come together, has therefore become a fundamental element of social welfare systems in various countries (Harris et al., 2016). However, it appears that these anticipated innovations, such as tailorized care services or smart solutions for administrative burdens, are not always realized in practice (Van Knippenberg, 2017). Previous studies, for instance, found that the relationships between functional diversity and team innovation were often initially insignificant and only significant under certain conditions such as participative leadership behaviors or open-mindedness (Mitchell & Boyle, 2015). More knowledge is therefore needed concerning the mechanism underlying the relationship between function diversity and team innovation in order to better understand why some functionally diverse teams do or do not realize their potential innovations (Mitchell & Boyle, 2015).

To address this knowledge gap, this study specifically builds on the theory of social categorization by studying social cohesion as a central mechanism. Social cohesion is an essential precondition for innovation as professionals need to work toward a shared goal and behave as “one” team to overcome challenges of trial and error, resistance, and uncertainty that are inherent in innovation (Hülsheger et al., 2009). Reasoning from the social categorization theory, scholars usually expect social cohesion to decrease as professional differences create alienation in the context of functionally diverse teams (Webber & Donahue, 2001). Recently though, there is growing support for an alternative interpretation that focuses on the fact that social categories are a perceptual cognitive construct (Haslam & Reicher, 2015). Continuing this line of reasoning, some scholars suggest that at a certain level of functional diversity, when everyone becomes different, the professionals may start to unite as one team again (Tekleab et al., 2016). The central question of this study is therefore as follows:

To what extent and in what manner does social cohesion mediate the relationship between functional diversity and team innovation?

To answer this research question, this study further explores the possibility of a curvilinear effect on team innovation (Hülsheger et al., 2009). More specifically, it is studied whether the relationship between functional diversity and team innovation is mediated by social cohesion in a way that, until a moderate level of functional diversity, there is a negative relationship between functional diversity and social cohesion, whereas beyond this threshold, functional diversity has a positive relationship with social cohesion. In turn, social cohesion is expected to have a positive relationship with team innovation. To examine these hypotheses, this study analyzed survey data among 887 professionals and 75 supervisors in 100 primary care teams in the Netherlands.

In doing so, this research contributes to the literature in at least two ways. First, this study contributes to research on functional diversity and team innovation by taking an alternative approach to the social categorization perspective that addresses their relationship. Three decades ago, Ashforth and Mael (1989) already noted that the complexity of identification in social categorization processes was insufficiently recognized, and yet, despite the substantial research effort, this question still arises today. Researchers are therefore advised to investigate an alternative approach to diversity in teams in curvilinear models (Mayo et al., 2017). By examining a U-shaped relationship between functional diversity and social cohesion, this study thus contributes to the overarching debate on interprofessional teams that has, so far, been dominated by conditional linear models. In addition, this study brings together the traditional and contemporary debate on innovation in interprofessional teams. On the one hand, by examining the mediating role of social cohesion, this study acknowledges the long-standing debate on the mechanism that translates the potential benefits of functional diversity into innovation. On the other hand, the introduction of a curvilinear relationship between functional diversity and social cohesion acknowledges the recently emerging debate on the nonlinear mechanism in interprofessional teams (Mitchell et al., 2022).

Second, this study contributes to the literature on social welfare by studying functionally diverse teams in the Dutch primary care context. It is known from previous academic work that the design of primary care teams influences the social welfare professionals as well as the citizens (e.g., see Stroebel et al., 2021). Looking at the functional diversity as a specific design characteristic, previous studies found that, for instance, teamwork with different professions resulted in improved tolerance of workload and better adherence of care (Norful et al., 2019). Because these insights about interprofessional teams in primary care are less advanced than in, for example, health care, an important contribution of this research is precisely in its application to the primary care context. Therefore, getting a better understanding of the primary care teams not only advances the literature but also offers insights for future policymaking.

The article is structured as follows. First, the article discusses relevant literature and proposes a conceptual model. Next, the methods and results of the analyses are presented. Finally, in the discussion and conclusions section, the findings are summarized and their implications for future research in primary care and for practitioners are discussed.

## Theoretical Framework

### Functional Diversity and Team Innovation

The crux of teamwork is that employees can “do better together” as they complement each other (Johnson et al., 2018). Organizations often incorporate this logic by bringing employees with different knowledge and skills together and thus create functionally diverse, also known as interprofessional or multidisciplinary, teams. The strength of these teams is in their wider pool of knowledge and skills that enrich their cognitive capacity (Lovelace et al., 2001). Furthermore, working in functionally diverse teams has been found to stimulate the team members to rethink existing approaches and generate a willingness to change (Perry-Smith, 2006). As such, functional diversity is seen as having a great potential for stimulating team innovation, which can be any new idea, process, or procedure that the team intentionally implemented (Van Knippenberg, 2017).

Interesting, however, is the fact that, based on previous empirical studies, the picture emerges that the relationship between functional diversity and team innovation is not as evident as expected (Anderson et al., 2014). The current understanding is therefore that functional diversity indeed has the potential to improve team innovation, yet these potential benefits are not realized automatically (Weiss et al., 2018).

### A Social Categorization Perspective on Functional Diversity

To gain a deeper understanding of what could hinder interprofessional teams from achieving greater team innovation in the first place, scholars commonly refer to the social categorization perspective, which argues that people have a natural tendency to classify themselves and others into social categories (Tajfel & Turner, 1979). This theorization furthermore explains that people tend to favor their “in-group” and attribute negative characteristics to the “out-groups.” These stereotypes and inaccurate attributions would then hinder people in identifying and communicating with those they perceive as part of an “out-group” (Tajfel & Turner, 1979).

The social categorization process is furthermore a cognitive process in a way that the perception of what is seen as different is shaped by the context (Ashforth & Mael, 1989). This means that, in the context of functional diversity, team members will initially tend to maintain and protect their own professional identity without engaging with different professions (Lovelace et al., 2001). Though when functional diversity increases further and relatively more different professionals work in a team, there are fewer comparable professions to identify with and everyone becomes different (Earley & Mosakowski, 2000). At this point, the professionals modify their perceptions on social categorizations by respecting differences and identify based on the shared goals that binds them (Gibson & Vermeulen, 2003), which suggests that the cohesion among the team members gets better again (Tekleab et al., 2016).

### The Mediating Role of Social Cohesion in the Relationship Between Functional Diversity and Team Innovation

A main implication of functional diversity, following the reasoning of the social categorization perspective, is thus its impact on the unity and strong social relationships among team members that reflects the social cohesion of a team (Keller, 2001). To illustrate, consider first a team with team members who share the same professional role, such as social workers. Their shared professional socialization, jargon, and procedures offer them a strong basis for social cohesion (Mitchell & Boyle, 2015). Now, consider a team with half of the professionals working as social workers and the other half working as pedagogues. Their professional difference makes them favor those who share the same professional role (i.e., “in-group”), as they struggle to identify and communicate with those who have a different professional role (i.e., “out-group”; Lovelace et al., 2001). The comparison of these two teams illustrates how an initial increase in functional diversity translates into less social cohesion through the process of social categorization.

However, teams can also be composed by numerous professional roles, such as social workers, psychologists, doctor’s assistants, municipal officials, pedagogues, disabled workers, and community nurses. As the alternative approach of social categorization theory emphasizes, the relatively large number of different professions makes it rather difficult for the professionals to find other team members with the same profession to identify with, changing the perception of social categories (Earley & Mosakowski, 2000). With this change of perspective, the professionals will focus on something else that binds them instead, such as their shared goals (Gibson & Vermeulen, 2003). Therefore, the additional comparison with this type of team demonstrates that, from a certain threshold, any further increase in the level of functional diversity makes the professionals feel united again (Tekleab et al., 2016). As such, this line of reasoning leads to the first hypothesis:

*Hypothesis 1: Functional diversity has a curvilinear relationship with social cohesion such that, until a certain level of functional diversity, the relationship is negative, however, after this threshold, the relationship becomes positive.*

The above then raises the question of how this explains the previous ambiguous findings regarding the relationship between function diversity and team innovation. The answer to this question lies in the fact that social cohesion creates a safe environment to share deviating information, critically reflect upon current approaches, and experiment (Edmondson, 1999). Furthermore, when facing resistance, which is inevitable when implementing innovations, members of social cohesive teams are more likely to persevere (West, 2002). Based on these arguments, it is no surprise that social cohesion is mentioned to be a critical precondition for innovation (Hülsheger et al., 2009). This leads to the following hypothesis:

*Hypothesis 2: Social cohesion has a positive relationship with team innovation.*

Following this line of reasoning, the functional diversity–team innovation relationship is thus hypothesized to evolve through social cohesion in such a way that the indirect relationship is U-shaped. This means that when functional diversity is either low or high, the team benefits of a high level of social cohesion based on profession or team membership, respectively, which spurs team innovation. In addition, teams with moderate levels of functional diversity have low levels of social cohesion because of social categorization based on professional identities, which hinders team innovation. The related hypothesis is as follows:

*Hypothesis 3: The relationship between functional diversity and team innovation is mediated by social cohesion such that, until a certain level of functional diversity, the mediated relationship is negative, however, after this threshold, the mediated relationship becomes positive.*

## Methods

### Functional Diversity in Primary Care Teams

The above hypotheses are studied in the context of the Dutch primary care sector. As in many high-income countries, the Dutch government decentralized the responsibility for primary care to the municipalities in 2015 (Harris et al., 2016). Since then, the municipalities work with locally oriented teams in which professionals share the responsibility for the social care of all citizens within a specific neighborhood (Van Rijn & Teeven, 2013). In essence, these teams are expected to provide integrated, cohesive care and support to improve the quality of social welfare (Van Rijn & Teeven, 2013). It is therefore the aim of these primary care teams to generate innovative solutions to the increasingly complex societal issues, and municipalities adopt different organizational strategies to achieve these innovations. A common strategy seen in many municipalities is to bring different professions, such as social workers, psychologists, municipal officials, pedagogues, disabled workers, and community nurses, together in the teams, which increases the level of functional diversity. Other municipalities introduce the role of a “generalist,” which means that the individual professional provides integrated services that would traditionally be provided by professionals with different expertise, and as a result, the team has low levels of functional diversity.

For this study, a total of five primary care organizations were selected through convenience sampling. These are all nonprofit organizations working in the three largest cities and the largest partnership of municipalities in the Netherlands based on the number of inhabitants. To reflect the various kinds of primary care teams in the Netherlands, each of the selected organizations have various target groups, like citizens of all ages, youth, adults, or citizens, with multiproblem cases.

Data were collected between July 2017 and May 2018, and the data collection took about 2 months in each organization. To avoid common source bias, the data were obtained from three different sources. Functional diversity was calculated from administrative data, social cohesion was measured through an online survey sent to 2,238 professionals working in 127 primary care teams, and team innovation was measured through an online survey targeted at the corresponding 83 supervisors, some of whom supervised multiple teams.

Before the data were collected, the terminology in the online surveys was adapted for each organization to reflect the terms used locally, such as “supervisor,” “team leader,” or “coach.” All respondents were further informed about the survey, and anonymity was guaranteed in the invitation e-mail. Two or three reminders were sent during the data collection period, which ultimately resulted in responses of 1,128 professionals (a response rate of 50%) and 79 supervisors (response rate of 95%). To ensure that the data were sufficiently representative, teams were only included in the final data set if at least 30% of team members and the team’s supervisor had completed the questionnaire. As a result, the final data set used to test the hypothesized model included 100 teams involving 887 professionals and 75 supervisors (81.1% of the teams in the population). The mean age of the professionals was 42 years (*SD* = 11), and 86% were female. The mean age of the supervisors was 47 years (*SD* = 8), and 77% were female. Further characteristics of the included respondents are presented in Table S1 of the supplementary file (http://links.lww.com/HCMR/A116).

### Measures

This section describes the measurement of the variables. The items comprising the measures are listed in the supplementary file (http://links.lww.com/HCMR/A117).

*Functional diversity* was measured using the administrative data on job titles. The job titles of the different organizations were compared, and similar job descriptions were grouped into an umbrella category, resulting in a total of 17 different categories. Examples of job titles are elderly worker, nurse, social worker, youth coach, psychologist, secretary, or generalist. The variety in job titles was computed using the normalized Blau index of heterogeneity, as suggested when an equal distribution of team members across the different categories is not always possible (Solanas et al., 2012). This means the Blau index, *B* = 1 − ∑*p*_i_^2^, is divided by the maximum value of the Blau index, *B*_max_ = *n*^2^(*k* − 1) + *a*(*a* − *k*)/(*kn*^2^). In these formulas, *p*_i_ is the relative frequency of categories, *k* is the number of categories, *n* is the group size, and *a* is calculated by *n* − *k* int[*n*/*k*] (Solanas et al., 2012). This resulted in minimum and maximum values of the normalized Blau index of 0 and 0.93, respectively, with an average heterogeneity of .64. The teams with a heterogeneity index of 0 consist exclusively of generalists, and the team with a heterogeneity index of .93 can be recognized by the many different specialists represented in the team.

*Social cohesion* was evaluated in the survey for the professionals using five items inspired by Carless and De Paola’s (2000) measurement scale for social cohesion. An example item is “Our team is united in trying to reach its goals for performance.” Responses were given on a 5-point Likert scale ranging from 1 = *strongly disagree* to 5 = *strongly agree*. The Cronbach’s alpha was .897.

*Team innovation* was assessed in the survey for the supervisors through four items based on the measurement scale of De Dreu (2002). An example item is “Team members often implement new ideas to improve the quality of our services.” Again, the responses were given on a 5-point Likert scale ranging from 1 = *strongly disagree* to 5 = *strongly agree*. Closer inspection revealed that the second question, which was the only one mirrored, did not fit well in the scale, also visible in the higher Cronbach alpha if this item was removed. The Cronbach alpha for the scale based on the remaining three items was .816.

*Control variables* in this study are team size, team stability, supervisors’ age and gender, and organizational affiliation. *Team size* is included in the study as a control variable because larger teams are expected to have more opportunities for team innovation (Hülsheger et al., 2009). On the other hand, however, in larger teams, the distance between team members is also greater, thereby hindering social integration and thus social cohesion (Smith et al., 1994). Team sizes were obtained from the organizations’ administration departments, and the average was 18 members with a standard deviation of 10 members, which is typical for primary care teams. *Team stability* is added as a control variable as more stable teams have been found to be more innovative (Van Engen & Van Woerkom, 2010) and cohesive (Dineen, 2005). Team stability was measured in the supervisor survey with a single item that asked to indicate what percentage of the team members had already joined the team a year ago. The four answer options ranged from 1 = *less than 25%* to 4 = *more than 75%*. The *age and gender of the supervisors* are also included as a control variable, as teams with older supervisors tend to benefit from a more innovative team climate (Jaiswal & Dhar, 2015) and female supervisors tend to show behavior that is positively related to team cohesion (Riisla et al., 2021). Lastly, to control for the potential influence of the different organizational contexts, dummy variables of the *organizational affiliation* have been included as well.

### Data Aggregation

To test the hypothesized model, the individuals’ scores for social cohesion had to be aggregated to the team level. To determine whether data aggregation is justified, the intraclass correlations (ICC1 and ICC2) and the within-group interrater reliability (*R*_wg_) were calculated (LeBreton & Senter, 2008). Given the relatively wide range of team sizes, the “average” team size (*Ng*) of 17,79 was calculated using the formula of Bliese and Halverson (1998, p. 168) to calculate the ICCs. This resulted in an ICC1 value of .12, which falls within the typical range of .05–.20, and an ICC2 value of .71, which is above the commonly used cutoff value of .7. Furthermore, the *R*_wg_ value of .78 is above the lower threshold of .7, and the corresponding *F* value is 3.47 and significant (Bliese & Halverson, 1998). On this basis, aggregating the individual social cohesion scores to the team level is justified.

### Descriptive Statistics and Correlations

The means, standard deviations, and correlations of the team constructs are presented in Table 1. All the correlations are below .7, and the variation inflation factors (reported in the supplementary files, Table S2, http://links.lww.com/HCMR/A118) are below 10, indicating that multicollinearity is not a concern. The correlations between functional diversity and social cohesion and between functional diversity and team innovation are both nonsignificant. The correlation between social cohesion and team innovation is positive and significant as expected, suggesting that more cohesive teams are also more innovative. Table 1 furthermore shows a significant and positive correlation between team size and functional diversity, and team innovation. This suggests that larger teams tend to be more functionally diverse and more innovative as has been seen in the meta-analysis by Hülsheger et al. (2009). The correlation between supervisors’ age and team innovation is also significant and positive, suggesting that teams with older supervisors are more innovative.

**Table 1:** Descriptive statistics and correlations (*n* = 100)

	Variable	Mean	*SD*	1	2	3	4	5	6	AVE/SIC
1	Functional diversity	0.64	0.32	—						
2	Social cohesion	3.96	0.41	.05	—					.77/.10
3	Team innovation	3.30	0.73	.11	.29**	—				.48/.10
4	Team size	18	10	.37**	−.16	.23*				
5	Team stability	3.4	0.75	−.15	.03	.01	−.02			
6	Supervisors’ age	48	9	−.12	.03	−.18	−.20*	−.05		
7	Supervisors’ gender	0.80		.07	.01	.23*	.14	−.05	−.11	

*Note.* AVE is the average of the squared factor loadings. SIC is the square of the correlation between the latent constructs (Farrell & Rudd, 2009). Dummy for supervisors’ gender is female. *p < .05, **p < .01.

To assess the convergent validity and the discriminant validity of the constructs, it was checked whether the Cronbach’s alphas were greater than .60 and that the average variance extracted was larger than the squared interconstruct correlation (Farrell & Rudd, 2009). Both these criteria were satisfied (see Table 1).

### Statistical Analysis

To test the hypothesized model, a structural equation model was calculated in AMOS following the two-step approach recommended by Anderson and Gerbing (1988). The first step consisted of testing the measurement reliability and validity by performing a confirmatory factor analysis of the measurement model. Following this, in the second step, the structural model with latent variables was tested. To assess whether the data fitted both the indicated measurement model and the structural model sufficiently well, goodness-of-fit statistics were evaluated. Indicators of a sufficient model fit are a nonsignificant χ^2^ value coupled with a χ^2^/*df* value of below 2, a goodness of fit (GFI) value of greater than .90, an RMSEA value of equal or less than .08, and comparative fit (CFI) and Tucker–Lewis indices (TLI) of greater than .90 (Brown, 2015).

### Measurement Model

In the first step, a confirmatory factor analysis was conducted to assess the convergent validity and discriminant validity of the constructs. All the standardized factor loadings were statistically significant (*p* < .01) and above the minimal cutoff value of .40 (Pituch & Stevens, 2016, p. 387). The initial fit of the measurement model was reasonable but did not satisfy all the criteria: χ^2^(26) = 74.64, *p* < .00, GFI = .844, RMSEA = .135, 90% CI [.10, .17[], TLI = .903, CFI = .930 (Table 2). To identify local misspecifications, any modification indexes of 3.84 or greater were further evaluated. Based on theoretical reasoning, an error term correlation was subsequently added between the fourth and fifth items of social cohesion that together represent the social dimension of social cohesion. The revised measurement model then provided a satisfactory model fit: χ^2^(25) = 30.25, *p* > .05, GFI = .936, RMSEA = .035, 90% CI [.00, .10], TLI = .989, CFI = .992 (Table 2).

**Table 2:** Goodness-of-fit test results for each model (*n* = 100)

	χ^2^ (*df*)	χ^2^/*df*	GFI	RMSEA	TLI	CFI
Measurement model						
Baseline model	705 (28)					
Theoretical model	74 (26)	2.87	.844	.135 (.10–.17)	.903	.930
Revised model	30 (25)	1.21	.936	.045 (.00–.10)	.989	.992
Structural model	139 (80)	1.74	.877	.087 (.06–.11)	.924	.960
Revised model	62 (37)	1.69	.904	.083 (.05–.12)	.962	.974
Criteria for good fit		≤2.00	>.9	<.08	>.9	>.9

### Structural Model

In the second step, the manifest independent variable functional diversity and the square of functional diversity, the control variables, and the different causal paths between the latent and the manifest variables were added to the measurement model to create the structural model. The structural model’s fit was satisfactory: χ^2^(80) = 139, *p* < .05, GFI = .877, RMSEA = .087, 90% CI [.06, .11], TLI = .924, CFI = .960 (Table 2). However, the results showed that none of the control variables had a significant influence on either social cohesion or team innovation, except for the influence of the supervisors’ age on team innovation. Therefore, to improve the structural model, the nonsignificant control variables were deleted.

**Figure 1:**
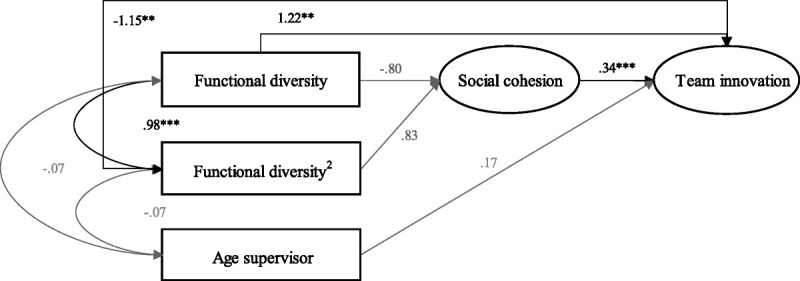
Structural model with standardized regression estimates.

The fit of the revised model was good, χ^2^(37) = 62, *p* < .05, GFI = .904, RMSEA = .083, 90% CI [.05, .12], TLI = .962, CFI = .974 (Table 2). The results, as shown in Figure 1, show a nonsignificant relationship between the square of functional diversity and social cohesion (β = .83, *p* = .07), rejecting the first hypothesis. Furthermore, the results show a significant positive relationship between social cohesion and team innovation (β = .42, *p* < .001), supporting the second hypothesis. In addition, the bootstrapped standardized indirect effect between the square of functional diversity and team innovation through social cohesion turns out to be insignificant (β = .28, bias-corrected 90% CI [−.02, .73], *p* = .11), which means that the third hypothesis is rejected.

### Additional Analyses

**Figure 2:**
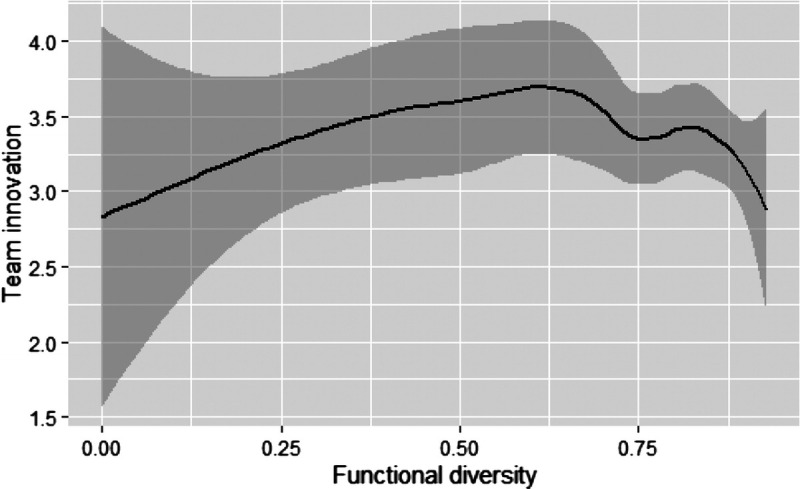
A plot of the relationship between functional diversity and team innovation. Note that the dark gray area shows the 95% confidence interval.

Figure 1 shows a significant relationship between the square of functional diversity and team innovation (β = −1.15, *p* < .01). This curvilinear relationship between functional diversity and team innovation is visualized using the ggplot2 package in R Studio in Figure 2 (Wickham, 2016). Figure 2 shows that moderate levels of functional diversity are associated with optimal levels of team innovation and low or high levels with minimal team innovation.

## Discussion and Conclusions

The aim of this study was to examine how functional diversity relates to team innovation by studying the mediating role of social cohesion. However, in contrast to the hypotheses, the results rejected this mediating role of social cohesion and the relationship between functional diversity and social cohesion. Instead, the results show that the relationship between functional diversity and team innovation evolves in an unexpectedly inverted U-shape. This means that, up to a certain degree of functional diversity, there is a positive relationship with team innovation. Confirming the information elaboration perspective, this positive relationship suggests that the different job roles come with a wider access to knowledge and skills, stimulating and enabling the team members to engage in collective problem-solving for team innovations (Van Knippenberg, 2017). However, the results also show that, from a certain degree of functional diversity, the relationship between functional diversity and team innovation becomes more complex and even turns negative. A potential explanation for this turning point could be that, at a certain point, there are too many different job roles, and the time and effort needed to create a mutual understanding is at the expense of the innovation potential (Keller, 2001).

An important implication of these findings is that the relationship between functional diversity and team innovation seems to be even more complex than theorized in the alternative approach to the social categorization theory. Nevertheless, this study also shows that social cohesion is an important predictor of team innovation, which underlines the need for a better understanding of how different job roles affect the cohesiveness among team members.

Although the findings of this study largely differ from the expectations, they still contribute to the literature in several ways. First, this study contributes to the literature on interprofessional teams and innovation by showing a curvilinear relationship between functional diversity and team innovation. As only a handful of researchers have found empirical evidence for a curvilinear influence of functional diversity (Tekleab et al., 2016), this study particularly contributed to the emerging literature stream that advocates a nonlinear approach to diversity (Mayo et al., 2017).

Second, this study contributes to the literature on primary care by showing that primary care teams with moderate levels of functional diversity benefit from relative high levels of team innovation. Although primary care scholars have devoted considerable research effort to unraveling the complexity of interprofessional teamwork, the potential curvilinear relationship between functional diversity and team innovation has remained virtually ignored in the primary care literature.

While making these contributions, this study also has limitations. A first limitation is in the generalizability of current findings to other contexts, as the Dutch primary care setting influences how professionals make sense of their diversity (Ashforth & Mael, 1989). Further research is therefore needed to examine to what extent the current findings can be replicated within other primary care contexts or beyond. A second limitation is in the research design. Although the current research design suits the research question, the design is also limited in a sense that the sample was too small to control for the fixed effects among organizations in a multilevel analysis technique. Furthermore, it lacks a thorough examination of the assumed perceptual cognitive social categorization process as it measures social cohesion as a proximal indicator. Future researchers would therefore do well to incorporate a multimethod research design, longitudinal studies and controlled experimental designs, that helps to gain a better understanding of the professionals’ sense-making of social categories when dealing with functional diversity.

Related to this, more research is also needed to understand the unexpected findings of this study. More specifically, the nonlinear relationship between functional diversity and team innovation, as demonstrated in this study, needs more solid theoretical progress. Researchers would therefore do well to look beyond existing theories to unravel the underlying mechanism through which team members of functionally diverse teams develop team innovations (Van Knippenberg, 2017). In this theoretical advancement, researchers are stimulated to continue to explore the role of social cohesion, as social cohesion appears to be a predictor of team innovation. A multilevel perspective that studies the role of stereotypes and the consequences for the behavior and mutual expectations of individual professionals, as explained by Van Dijk et al. (2017), seems particularly promising. In addition, it would be interesting to gain a more in-depth understanding of the context that shapes the relationship between functional diversity and social cohesion and team innovation by, for example, studying the role of the leadership of the team leader (e.g., see Mitchell & Boyle, 2019).

### Practice Implications

This study also has relevant implications for policymakers and supervisors in primary care organizations. Policymakers are advised to carefully consider the level of functional diversity when designing primary care teams. Based on the current findings, they should best avoid either too low or high levels of functional diversity to optimize the team innovation. Of course, the level of functional diversity in a team also needs to be considered in the broader context, as the expertise of the professionals ideally matches the needs of the citizens. Moreover, policymakers are advised to assess to what extent team innovation is indeed favorable for the professionals and citizens, as team innovation risks to increase workload or leads to a lack of clarity over team goals (Janssen et al., 2004). The supervisors of the primary care teams need to be aware that, although it is uncertain how social cohesion is developed among professionals with different job roles, social cohesion is an important predictor of team innovation. Supervisors who want to increase team innovations would therefore do well to achieve at least a minimum level of social cohesion, for example, by increasing team autonomy (van Zijl et al., 2019).

In summary, this study reveals some interesting findings suggesting that, at moderate levels of functional diversity, different job roles stimulate and enable to create more team innovation. It seems furthermore beneficial to create high levels of social cohesion as this is positively related to team innovation. More research is needed to understand how social cohesion is influenced by the different job roles in a team, as the expected curvilinear relationship appears to be insignificant.

## Ethical Approval

The study is based on an anonymous survey that is free from radical, incriminating, or intimate questions. All participants contributed voluntary and were considered to be competent to fill in the survey in a reasonable time period of approximately 20 minutes. Complete confidentiality and anonymity were guaranteed, and all participants completed the consent form. The data were managed in accordance with the Dutch Personal Data Protection Act. Therefore, at the time the study was conducted, ethical approval was not required by the research institute nor by the Dutch law on medical research (Medical Research Involving Human Subjects Act, http://www.ccmo.nl).

## Supplementary Material
