# Supplementary material for: Functional diversity and team innovation: A study on the mediating role of social cohesion in primary care teams
Source: Health Care Manage Rev. 2023 Mar 27;48(3):229–36. doi: 10.1097/HMR.0000000000000369 (PMC10227926; doi:10.1097/HMR.0000000000000369)
Supplement: Supplementary file 2 [file hcmr-48-229-s002.docx]

SUPPLEMENTARY FILE

Table S1. Descriptive statistics and participant characteristics

|  | Variable | Mean | S.D. | Minimum | Maximum | N |
| --- | --- | --- | --- | --- | --- | --- |
| 1 | Functional heterogeneity^3^ | .64 | .32 | .0 | .93 | 100 |
| 2 | Team cohesion^1^ | 3.97 | .41 | 2.80 | 4.70 | 100 |
| 3 | Team innovation^2^ | 3.32 | .73 | 1.33 | 5.00 | 100 |
| 4 | Team size | 18 | 10 | 6 | 51 | 100 |
| 5 | Female (dummy: yes)^1^ | .86 |  | 0 | 1 | 885 |
| 6 | Age in years^1^ | 42 | 11 | 21 | 65 | 879 |
| 7 | Female (dummy: yes)^2^ | .77 |  | 0 | 1 |  |
| 8 | Age in years^2^ | 47 | 8 | 31 | 63 | 75 |

^1^ Professionals; ^2^ Supervisors; ^3^ Administrative data

Table S2. Collinearity Statistics

|  | Variable | tolerance | VIF |
| --- | --- | --- | --- |
| 1 | Functional heterogeneity | .81 | 1.23 |
| 2 | Team cohesion | .96 | 1.04 |
| 3 | Team size | .79 | 1.27 |
| 4 | Team stability | .96 | 1.04 |
| 5 | Supervisor age | .95 | 1.06 |
| 6 | Supervisor gender | .95 | 1.06 |

Note. Tolerance values below .1 and Variance Inflation Factor (VIF) values

above 10 are indicative of serious multicollinearity problems

Items used

Team Cohesion

1. Our team is united in trying to reach its goals for performance.
2. We all take responsibility for setbacks or poor team performance.
3. In our team we help each other to complete tasks.
4. In our team we get along well together.
5. In our team we can trust each other.

Team innovation

1. Team members often implement new ideas to improve the quality of our services.
2. This team gives little consideration to new and alternative methods and procedures for doing their work. (reverse coded)*
3. Team members often produce new methods or procedures.
4. This team is an innovative team.

*This item was deleted
